# Author Correction: Somatic mosaicism and common genetic variation contribute to the risk of very-early-onset inflammatory bowel disease

**DOI:** 10.1038/s41467-022-31010-2

**Published:** 2022-06-22

**Authors:** Eva Gonçalves Serra, Tobias Schwerd, Loukas Moutsianas, Athena Cavounidis, Laura Fachal, Sumeet Pandey, Jochen Kammermeier, Nicholas M. Croft, Carsten Posovszky, Astor Rodrigues, Richard K. Russell, Farah Barakat, Marcus K. H. Auth, Robert Heuschkel, Matthias Zilbauer, Krzysztof Fyderek, Christian Braegger, Simon P. Travis, Jack Satsangi, Miles Parkes, Nikhil Thapar, Helen Ferry, Julie C. Matte, Kimberly C. Gilmour, Andrzej Wedrychowicz, Peter Sullivan, Carmel Moore, Jennifer Sambrook, Willem Ouwehand, David Roberts, John Danesh, Toni A. Baeumler, Tudor A. Fulga, Eli M Carrami, Ahmed Ahmed, Rachel Wilson, Jeffrey C. Barrett, Abdul Elkadri, Anne M. Griffiths, COLORS in IBD group investigators, Marlen Zurek, Caterina Strisciuglio, Mamoun Elawad, Bernice Lo, Oxford IBD cohort study investigators, Carolina Arancibia-Carcamo, Adam Bailey, Ellie Barnes, Elizabeth Louise Bird-Lieberman, Oliver Brain, Barbara Braden, Jane Collier, James East, Lucy Howarth, Satish Keshav, Paul Klenerman, Simon Leedham, Rebecca Palmer, Fiona Powrie, Alison Simmons, INTERVAL Study, Matthew Walker, Zoe Tolkien, Stephen Kaptoge, David Allen, Susan Mehenny, Jonathan Mant, Emanuele Di Angelantonio, Simon G. Thompson, Swiss IBD cohort investigators, Bahtiyar Yilmaz, Pascal Juillerat, Markus Geuking, Reiner Wiest, Andrew J. Macpherson, Francisco Damian Bravo, Lukas Brügger, Ove Carstens, Ulrike Graf Bigler, Benjamin Heimgartner, Monica Rusticeanu, Sybille Schmid-Uebelhart, Bruno Strebel, Aurora Tatu, Radu Tutuian, Reiner Wiest, Ove Øyås, Charlotte Ramon, Jörg Stelling, Yannick Franc, Nicolas Fournier, Valerie E. H. Pittet, Bernard Burnand, Mara Egger, Yannick Franc, Delphine Golay, Astrid Marot, Leilla Musso, Valérie Pittet, Jean-Benoît Rossel, Vivianne Seematter, Joachim Sommer, Rachel Vulliamy, Pierre Michetti, Michel H. Maillard, Céline Keller, Michel H. Maillard, Andreas Nydegger, Alain Schoepfe, Eva Archanioti, Jessica Ezri, Montserrat Fraga, Alain Schoepfer, Christoph Müller, Gerhard Rogler, Luc Biedermann, Mirjam Blattmann, Sabine Burk, Barbara Dora, Michael Fried, Benjamin Misselwitz, Beat Müllhaupt, Nicole Obialo, Daniel Pohl, Nadia Raschle, Gerhard Rogler, Michael Scharl, Stephan Vavricka, Roland Von Känel, Jonas Zeitz, Karim Abdelrahman, Gentiana Ademi, Jan Borovicka, Stephan Brand, Remus Frei, Johannes Haarer, Christina Knellwolf-Grieger, Claudia Krieger-Grübel, Patrizia Künzler, Christa Meyenberger, Pamela Meyer, Nina Röhrich, Mikael Sawatzki, Martin Schelling, Gian-Marco Semadeni, Michael Sulz, Dorothee Zimmermann, Patrick Aepli, Dominique H. Criblez, Cyrill Hess, Jean-Pierre Richterich, Johannes Spalinger, Dominic Staudenmann, Andreas Stulz, Stefanie Wöhrle, Amman Thomas, Claudia Anderegg, Henrik Köhler, Rachel Kusche, Anca-Teodora Antonino, Eviano Arrigoni, José M. Bengoa, Sophie Cunningham, Philippe de Saussure, Laurent Girard, Diana Bakker de Jong, Polat Bastürk, Simon Brunner, Lukas Degen, Petr Hruz, Carolina Khalid-de Bakker, Jan Niess, Bruno Balsiger, Janine Haldemann, Gaby Saner, Frank Seibold, Peter Bauerfeind, Andrea Becocci, Dominique Belli, Janek Binek, Peter Hengstler, Stephan Boehm, Tujana Boldanov, Patrick Bühr, Rebekka Koller, Vanessa Rueger, Arne Senning, Emanuel Burri, Sophie Buyse, Dahlia-Thao Cao, Fabrizia D’Angelo, Joakim Delarive, Christopher Doerig, Roxane Hessler, Claudia Preissler, Ronald Rentsch, Branislav Risti, Marc Alain Ritz, Michael Steuerwald, Jürg Vögtlin, Markus Sagmeister, Bernhard Sauter, Susanne Schibli, Christiane Sokollik, Johannes Spalinger, Hugo Schlauri, Jean-François Schnegg, Mariam Seirafi, Holger Spangenberger, Philippe Stadler, Peter Staub, Volker Stenz, Michela Tempia-Caliera, Joël Thorens, Kaspar Truninger, Patrick Urfer, Francesco Viani, Dominique Vouillamoz, Silvan Zander, Tina Wyli, UK IBD Genetics Consortium, L. Jostins, N. A. Kennedy, T. Ahmad, C. A. Lamb, C. Edwards, A. Hart, C. Hawkey, J. C. Mansfield, C. Mowat, W. G. Newman, A. Simmons, M. Tremelling, J. C. Lee, N. J. Prescott, C. G. Mathew, C. W. Lees, NIDDK IBD Genetics Consortium, D. P. B. McGovern, S. R. Targan, G. Botwin, E. Mengesha, P. Fleshner, C. Landers, D. Li, J. D. Rioux, A. Bitton, J. Côté-Daigneault, M. J. Daly, R. Xavier, K. Morris, G. Boucher, J. H. Cho, C. Abraham, M. Merad, B. Sands, I. Peter, K. Hao, Y. Itan, R. H. Duerr, L. Konnikova, M. B. Schwartz, S. Proksell, E. Johnston, V. Miladinova, W. Chen, S. R. Brant, L. Datta, M. S. Silverberg, L. P. Schumm, S. Birch, M. Giri, K. Gettler, Y. Sharma, C. Stevens, M. Lazarev, T. Haritunians, Scott B. Snapper, Neil Shah, Aleixo M. Muise, David C. Wilson, Holm H. Uhlig, Carl A. Anderson

**Affiliations:** 1 Wellcome Sanger Institute, Wellcome Genome Campus, Hinxton, UK; 2 Translational Gastroenterology Unit, University of Oxford, Oxford, UK; 3 Great Ormond Street Hospital, London, UK; 4 Blizard Institute, Barts and the London School of Medicine, Queen Mary University of London, London, UK; 5 The Royal London Children’s Hospital, Barts Health NHS Trust, London, UK; 6 Universitätsklinikum, Ulm, Germany; 7 Department of Paediatrics, University of Oxford, Oxford, UK; 8 Royal Hospital for Children, Glasgow, UK; 9 Alder Hey Children’s Hospital, Liverpool, UK; 10 Addenbrooke’s Hospital, Cambridge, UK; 11 Department of Paediatrics, Gastroenterology and Nutrition, Jagiellonian University Medical College, Krakow, Poland; 12 Division of Gastroenterology and Nutrition and Children’s Research Center, University Children’s Hospital Zurich, Zurich, Switzerland; 13 Institute of Genetics and Molecular Medicine, University of Edinburgh, Scotland, UK; 14 IBD Research Unit, Department of Gastroenterology, Addenbrooke’s Hospital, Cambridge, UK; 15 NIHR Blood and Transplant Research Unit in Donor Health and Genomics, Department of Public Health and Primary Care, University of Cambridge, Cambridge, UK; 16 INTERVAL Coordinating Centre, Department of Public Health and Primary Care, University of Cambridge, Cambridge, UK; 17 Department of Haematology, University of Cambridge, Cambridge, UK; 18 NHS Blood and Transplant - Oxford Centre, Level 2, John Radcliffe Hospital, Oxford, UK; 19 Biomedical Research Centre, Oxford – Haematology Theme, Radcliffe Department of Medicine, University of Oxford, John Radcliffe Hospital, Oxford, UK; 20 Weatherall Institute of Molecular Medicine and the Radcliffe Department of Medicine, University of Oxford, John Radcliffe Hospital, Oxford, UK; 21 National Institute of Health Research Oxford Biomedical Research Centre, Surgical Innovation and Evaluation and Molecular Diagnostics Themes, University of Oxford, Oxford, UK; 22 Department of Biochemistry and Pediatrics, Faculty of Medicine, University of Toronto, Toronto, ON Canada; 23 SickKids Inflammatory Bowel Disease Centre and Cell Biology Program, Research Institute, Hospital for Sick Children, Toronto, ON Canada; 24 Division of Gastroenterology, Hepatology and Nutrition, Boston Children’s Hospital, Boston, MA USA; 25 Harvard Medical School, Boston, MA USA; 26 Division of Gastroenterology, Brigham and Women’s Hospital, Boston, MA USA; 27 Child Life and Health, University of Edinburgh, Edinburgh, UK; 28 St. Georg Hospital, Leipzig, Germany; 29 Department of Translational Medical Sciences, Section of Pediatrics, University of Naples, Naples, Italy; 30 Sidra Medical and Research Center, Doha, Qatar; 31 NHS Blood and Transplant, Longley Lane, Sheffield, UK; 32 Maurice Müller Laboratories, Department for Biomedical Research, University of Bern, Bern, Switzerland; 33 Department of Visceral Surgery and Medicine, Bern University Hospital, University of Bern, Bern, Switzerland; 34 Department of Biosystems Science and Engineering and SIB Swiss Institute of Bioinformatics, ETH Zurich, Basel, Switzerland; 35 Institute of Social and Preventive Medicine (IUMSP), Lausanne University Hospital, Lausanne, Switzerland; 36 Gastroenterology La Source-Beaulieu, Lausanne, Switzerland; 37 Service of Gastroenterology and Hepatology, Department of Medicine, Centre Hospitalier Universitaire Vaudois and University of Lausanne, Lausanne, Switzerland; 38 Division of Experimental Pathology, Institute of Pathology, University of Bern, Bern, Switzerland; 39 Department of Gastroenterology and Hepatology, University Hospital Zurich, University of Zurich, Zurich, Switzerland; 40 Clinique de Montchoisi, Lausanne, Switzerland; 41 Kantonsspital St-Gallen, St. Gallen, Switzerland; 42 Kantonsspital Luzern, Luzern, Switzerland; 43 GI private practice, Waldkirch, St. Gallen, Switzerland; 44 Kantonspital Aarau, Klinik für Kinder und Jugendliche, Aarau, Switzerland; 45 Hôpital Riviera—Site du Samaritain, Vevey, Vaud, Switzerland; 46 GI private practice, Geneva, Switzerland; 47 Department of Gastroenterology and Hepatology, Basel University Hospital, Basel, Switzerland; 48 Gastroenterologische Praxis, Bern, Switzerland; 49 Department Gastroenterology and Hepatology, Stadtspital Triemli, Zurich, Switzerland; 50 Department of Pediatric, Geneva University Hospital, Geneva, Switzerland; 51 Gastroenterologie am Rosenberg, St. Gallen, Switzerland; 52 Spital Bülach, Bülach, Zurich, Switzerland; 53 Department of Biomedicine, University of Basel, Basel, Switzerland; 54 Department Gastroenterology, Kantonsspital Liestal, Liestal, Switzerland; 55 GI private practice, Yverdon-les-Bains, Liestal, Switzerland; 56 Hôpital Neuchâtelois, La Chaux-de-fonds, Neuchâtel, Switzerland; 57 Department Gastroenterology and Hepatology, Geneva University Hospital, Geneva, Switzerland; 58 GI private practice, Lausanne, Switzerland; 59 Clinique Cecil, Lausanne, Switzerland; 60 Kantonsspital Olten, Olten, Switzerland; 61 GI Private Practice, St. Gallen, Switzerland; 62 GI Practice, Dietikon, Switzerland; 63 GI Practice, Liestal, Switzerland; 64 GI Private Practice, Heerbrugg, Switzerland; 65 Klinik Hirslanden Zürich, Zurich, Switzerland; 66 Kinderklinik Bern, Bern University Hospital, Bern, Switzerland; 67 Derby Center, Wil, Switzerland; 68 GI Private Practice, Montreux, Switzerland; 69 Clinique La Colline, Geneva, Switzerland; 70 Kantonsspital Wolhusen, Wolhusen, Switzerland; 71 GI Private Practice, Payerne, Switzerland; 72 Spital Heiden Appenzell Ausserrhoden, Heiden, Switzerland; 73 Kantonsspital Münsterlingen, Münsterlingen, Switzerland; 74 Clinique des Grangettes, Chêne-Bougeries, Switzerland; 75 GI Private Practice, Yverdon, Switzerland; 76 GI Private Practice, Langenthal, Switzerland; 77 Hirslanden Klinik Aarau, Gastro Zentrum, Aarau Switzerland; 78 Private Practice, Vevey, Switzerland; 79 Private Practice, Pully, Switzerland; 80 Spital Limmattal, Schlieren, Switzerland; 81 Infirmière de Recherche chez CHUV Lausanne University Hospital, Lausanne, Switzerland; 82 The Wellcome Trust Centre for Human Genetics, Oxford, UK; 83 Precision Medicine Exeter, University of Exeter, Exeter, UK; 84 Institute of Cellular Medicine, Newcastle University, Newcastle Upon Tyne, UK; 85 Department of Gastroenterology, Torbay Hospital, Torbay, UK; 86 Department of Medicine, St. Mark’s Hospital, Harrow, UK; 87 Nottingham Digestive Diseases Centre, Queens Medical Centre, Nottingham, UK; 88 Institute of Human Genetics, Newcastle University, Newcastle upon Tyne, UK; 89 Department of Medicine, Ninewells Hospital and Medical School, Dundee, UK; 90 Genetic Medicine, Manchester Academic Health Science Centre, Manchester, UK; 91 Gastroenterology & General Medicine, Norfolk and Norwich University Hospital, Norwich, UK; 92 Department of Medical and Molecular Genetics, King’s College London School of Medicine, Guy’s Hospital London, London, UK; 93 Gastrointestinal Unit, Western General Hospital University of Edinburgh, Edinburgh, UK; 94 F. Widjaja Inflammatory Bowel and Immunobiology Research Institute, Cedars-Sinai Medical Center, Los Angeles, CA USA; 95 Montreal Heart Institute, Research Center, Montreal, Canada; 96 McGill University Health Centre, Montreal, Quebec Canada; 97 Centre Hospitalier de l’Université de Montréal, Montreal, Canada; 98 Institute of Molecular Medicine Finland, Helsinki, Finland; 99 Broad Institute of MIT, Cambridge, Massachusetts USA; 100 Charles Bronfman Institute for Personalized Medicine, Departments of Medicine and Genetics, Icahn School of Medicine, New York, USA; 101 Department of Medicine, Yale University, New Heaven, USA; 102 Precision Immunology Institute, Icahn School of Medicine at Mount Sinai, New York, USA; 103 Department of Medicine, Icahn School of Medicine at Mount Sinai, New York, USA; 104 Department of Medicine, University of Pittsburgh School of Medicine, Pittsburgh, PA USA; 105 Department of Medicine, Rutgers University, New Brunswick, USA; 106 Samuel Lunenfeld-Tanenbaum Research Institute, Mount Sinai Hospital, University of Toronto, New York, USA; 107 Department of Public Health Sciences, University of Chicago, Chicago, USA; 108 Department of Medicine, Johns Hopkins University School of Medicine, Baltimore, MD USA; 109 Present Address: Dr. von Hauner Children’s Hospital, Department of Pediatrics, University Hospital, Ludwig Maximilians University, Munich, Germany

**Keywords:** Immunogenetics, Medical genetics, Inflammatory bowel disease, Immunological deficiency syndromes

Correction to: *Nature Communications* 10.1038/s41467-019-14275-y, published online 21 February 2020.

Since the publication of this work, Eli M Carrami has changed their name from Mohammad Karaminejadranjbar. This has now been amended in the HTML and PDF versions of the article.

