# Supplementary material for: Somatic mosaicism and common genetic variation contribute to the risk of very-early-onset inflammatory bowel disease
Source: Nat Commun. 2020 Feb 21;11:995. doi: 10.1038/s41467-019-14275-y (PMC7035382; doi:10.1038/s41467-019-14275-y)
Supplement: Supplementary file 1 — Supplementary Information [file 41467_2019_14275_MOESM1_ESM.pdf]

Somatic mosaicism and common genetic variation contribute to the risk of very-early-onset inflammatory bowel disease

Gonçalves Serra et al

SUPPLEMENTARY NOTE 1

CYBB patient summary: The male patient first developed gastrointestinal diarrhoea and rectal bleeding at age 4 weeks (**Supplementary Figure 5A**). The symptoms were attributed to cow's milk protein allergy-associated colitis. A cow's milk- and egg-free diet improved symptoms, but episodes of diarrhoea remained. At two years of age, further episodes or rectal bleeding triggered referral to paediatric gastroenterology; endoscopy suggested the diagnosis of ulcerative colitis. Multiple subsequent endoscopies over the succeeding (33) years reported pancolitis with deep ulcerations (**Supplementary Figure 5A**), with histological features of lamina propria inflammation, crypt abscesses, mild crypt architectural distortion and goblet cell depletion consistent with a diagnosis of ulcerative colitis. Poorly formed granulomas were reported in some biopsies. On one occasion backwash ileitis was identified. In view of a pilonidal abscess at age 2, several perianal and axillary abscesses and an anterior fistula at age 10 (treated by fistulotomy and seton insertion), a clinical diagnosis of Crohn's disease was considered, although the endoscopies and histopathology were more in keeping with ulcerative colitis.

Inflammation was difficult to control, with multiple episodes of intestinal and skin inflammation, as indicated by increased CRP or ESR (**Supplementary Figures 5C-D**). In contrast, neutrophil, lymphocyte and platelet counts were largely normal over time (**Supplementary Figures 5E-G**).

Treatment included exclusive enteral nutrition as a child, as well as oral and systemic steroids (hydrocortisone, prednisolone, steroid enemas) with immunomodulators (azathioprine, mercaptopurine, methotrexate) as well as aminosalicylate anti-inflammatories (mesalazine, sulfasalazine). Anti-TNF therapy controlled inflammation over a period of years, but was stopped after a community acquired pneumonia (age 33 years). Anti-$\alpha_4\beta_7$ integrin therapy (vedolizumab) was started at age 34 years of age.

Cutaneous features included apparent eczema in infancy. Subsequently severe, ill-defined skin inflammation developed, involving face, scalp and extremities, including axillae, elbow, knee, and finger. After multiple independent dermatology specialist opinions and multiple biopsies, the skin reaction was termed folliculitis, hidriasis supportiva as well as psoriasiform. Nevertheless, different

diagnoses over time illustrate the difficult classification (differential diagnosis included eczematoid lesions, fixed urticaria, follicular type of seborrhoeic dermatitis, dermatitis herpetiformis, contact dermatitis, discoid lupus, cutaneous vasculitis and lichenoid reaction). Adverse drug responses were also considered, especially in relation to methotrexate and anti-TNF therapy. At one stage immunofluorescence showed a linear band of IgM at the dermoepidermal junction. Treatment included topical and systemic therapy with steroids, antibiotics and antiseptic medications such as fusidic acid, ampicillin, flucloxacillin, erythromycin, minocyline and cephalosporins, or antifungal treatments. This illustrates the degree of overlap between different gut and cutaneous disorders, as well the diagnostic uncertainty created by this somatic mosaicism.

The patient presented with middle ear infection in childhood, and pneumonia as an adult. Immunological tests did show normal immunoglobulin levels (IgG, IgA, IgM and IgE), normal lymphocyte subsets and absence of autoantibodies (including ANTI anti-nuclear, anti-smooth muscle, anti-mitochondrial, and anti-neutrophil antibodies).

SUPPLEMENTARY NOTE 2

Likely benign and variants of uncertain significance (VUS) identified in VEO-IBD patients: The initial screening for variants in known IBD-associated Mendelian disorder genes led to the identification of three likely benign variants and two variants of uncertain significance in a total of five VEO-IBD patients.

Likely benign variants:

- ***CYBB:*** In a female presenting with severe perianal disease, prominent granulomas and multiple infections, a heterozygous *CYBB* defect was identified (p.D447V). This variant is predicted to be damaging by both SIFT and PolyPhen-2, has a very low frequency in ExAC (AF < 0.05%), is not observed in the INTERVAL control exomes and the ancestral allele is highly conserved throughout evolution. Since non-random X inactivation in *CYBB* may be associated with IBD or immune defects[1,2] we performed a functional analysis via the DHR assay and excluded this possibility in the patient.
- ***NLRC4:*** The heterozygous missense p.G786V variant in *NLRC4* was shared by two VEO-IBD patients, but was also present in eight INTERVAL and 290 ExAC individuals.

- ***WAS:*** The hemizygous p.E131K missense variant identified in *WAS* has an AF of almost 1% in European non-Finnish ExAC individuals (AF=0.0085), including five hemizygous males.

The discovery of presumed healthy individuals carrying these *disruptive* alleles significantly decreases our confidence that such variants are fully penetrant, causal VEO-IBD variants and thus we define them as likely benign events.

VUS variants:

- ***NCF2:*** The homozygous p.G501R variant, occurring in the SH3 domain of *NCF2* (encoding p67phox), is extremely rare in ExAC (MAF ~ 0.001%; no homozygotes), but a heterozygous VEO-IBD carrier has previously been reported[3]. Functional assays in the previously reported patient confirmed reduced interaction between p47phox and p.G501R p67phox, compared to wild-type p67phox, suggesting a functionally relevant effect caused by this amino acid change[3]. However, since NADPH oxidase activity was not validated in our current patient, and to our knowledge not in the literature either, we took the conservative approach of classifying this variant as of unknown significance.
- ***NLRC4:*** p.C258R is novel and thus not described in any reference dataset. Gain-of-function *de novo* missense mutations within the nucleotide-binding domain (NBD) of *NLRC4* are established causes of autoinflammatory enterocolitis phenotypes[4,5]. Parental data was not available for us to determine if the variant detected in this patient constituted a *de novo* event. Furthermore, because the *NLRC4* p.C258R variant occurs outside the NBD region, and the patient did not present with an autoinflammatory phenotype, we defined this allele as a variant of uncertain significance.

SUPPLEMENTARY FIGURES

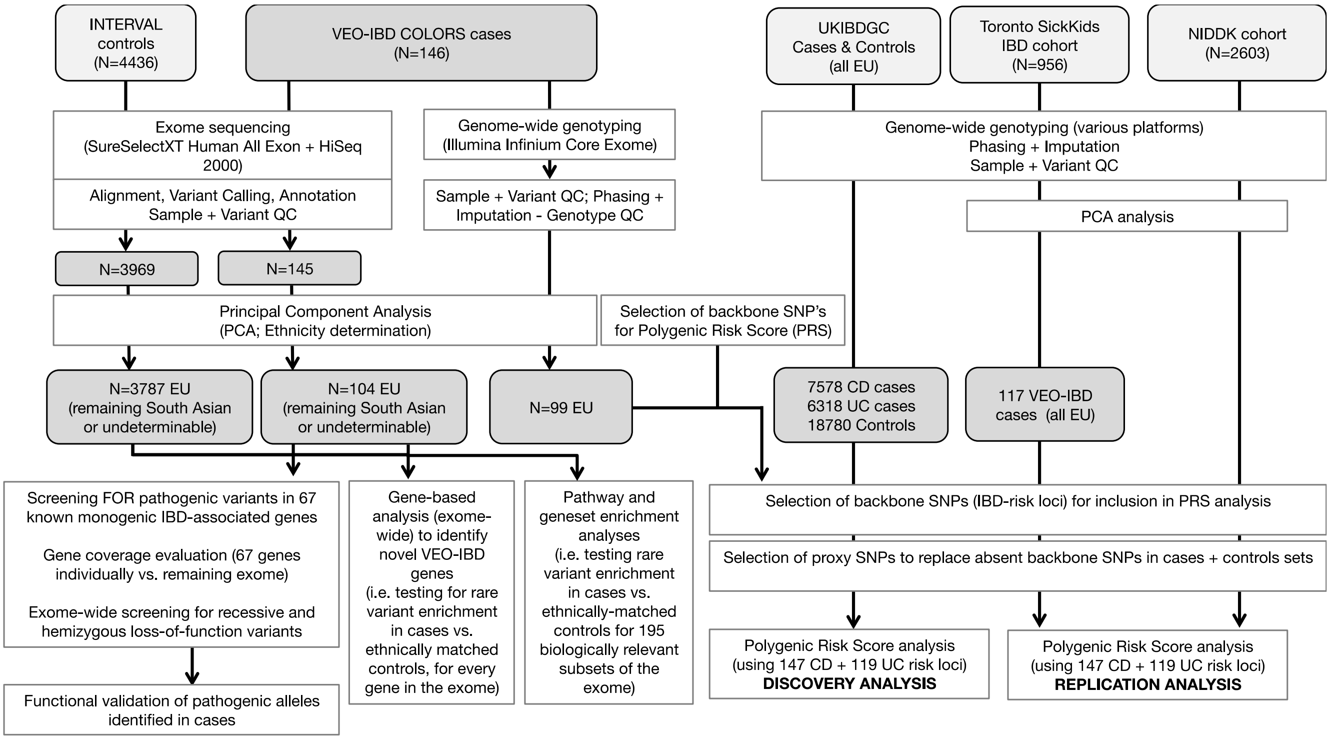

Supplementary Figure 1. Overview of study workflow and investigative analyses. Outline of exome and genotype-based analyses included in this study, accounting for rare or common genetic variation in VEO-IBD cases, respectively. A replication experiment to validate the initial polygenic risk score results was conducted using an additional set of VEO-IBD cases (Toronto SickKids, N=117) and population controls (NIDDK Genetics Consortium, N= 2,603).

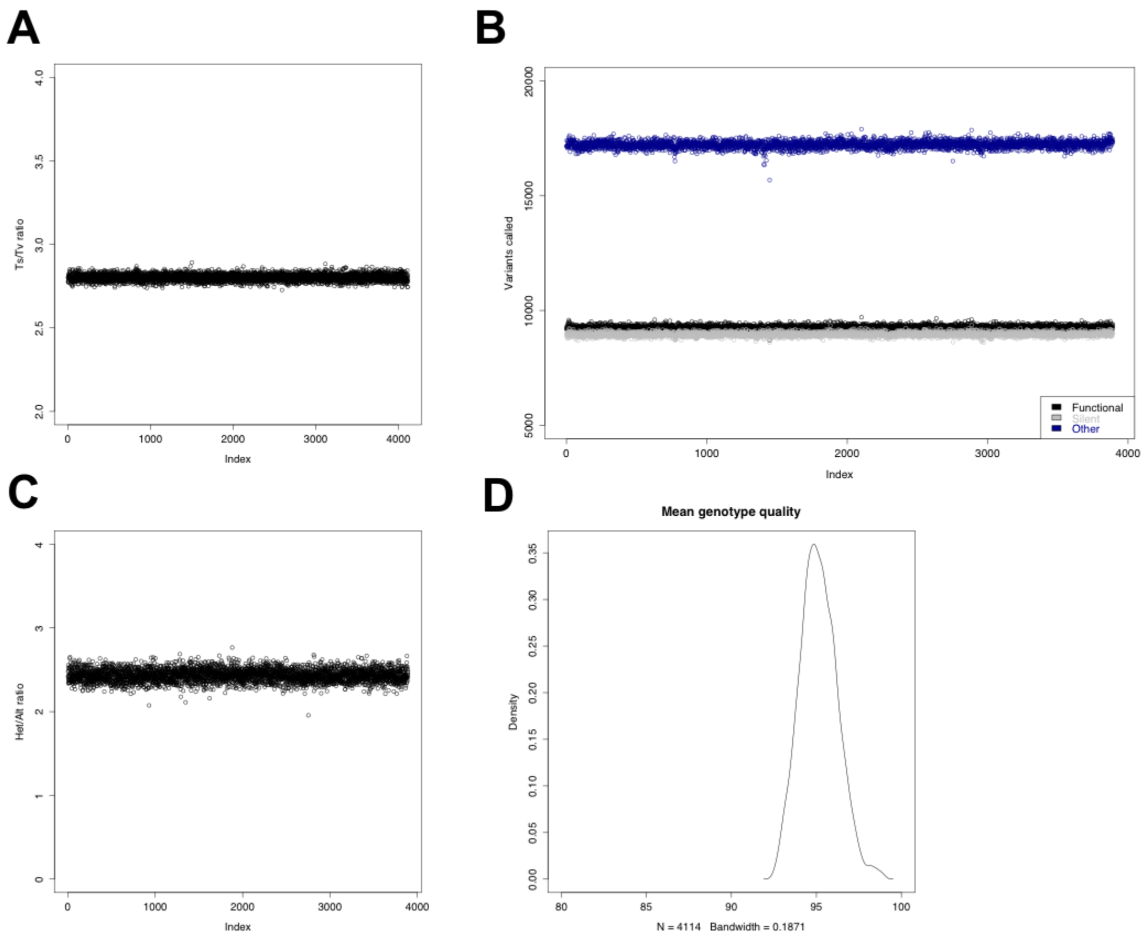

Supplementary Figure 2. Sequencing quality assessment post quality control in VEO-IBD cases and INTERVAL control exome samples. A) Transition to transversion (Ts/Tv) ratio per sample. **B)** Number of variants called according to functional consequence estimated using Ensembl VEP version 75. Functional variants include: stop gained, splice donor and acceptors, frameshift, missense, inframe insertion and deletions, initiator codon and splice region variants. Silent variants include those that are synonymous. Other variants include intronic and variants in 5’ or 3’ untranslated regions (UTRs). **C)** Heterozygous to homozygous-alternative-allele (Het/Alt) ratio per sample. **D)** Distribution of mean genotype quality (GQ) per sample. Genotype quality given by Haplotype Caller version 3.4. Overall, samples are consistent with each other with respect to these QC metrics.

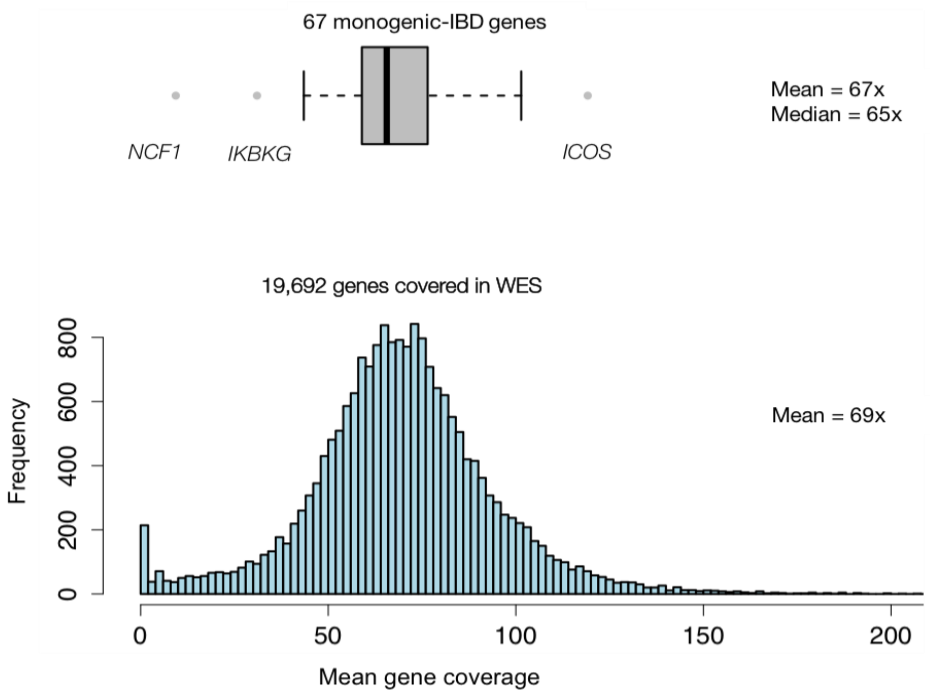

Supplementary Figure 3. Sequencing coverage of mendelian-IBD genes compared with the rest of the exome. Sequencing coverage was calculated exome-wide, at each gene, by extracting the read depth at each nucleotide (within exonic sequences of each gene); this was conducted on a per-sample level. The read depth was then averaged per-coordinate across samples to produce an average capture per position, as well as a mean and median coverage per gene. QC pass VEO-IBD cases (N=145) were used in this analysis.

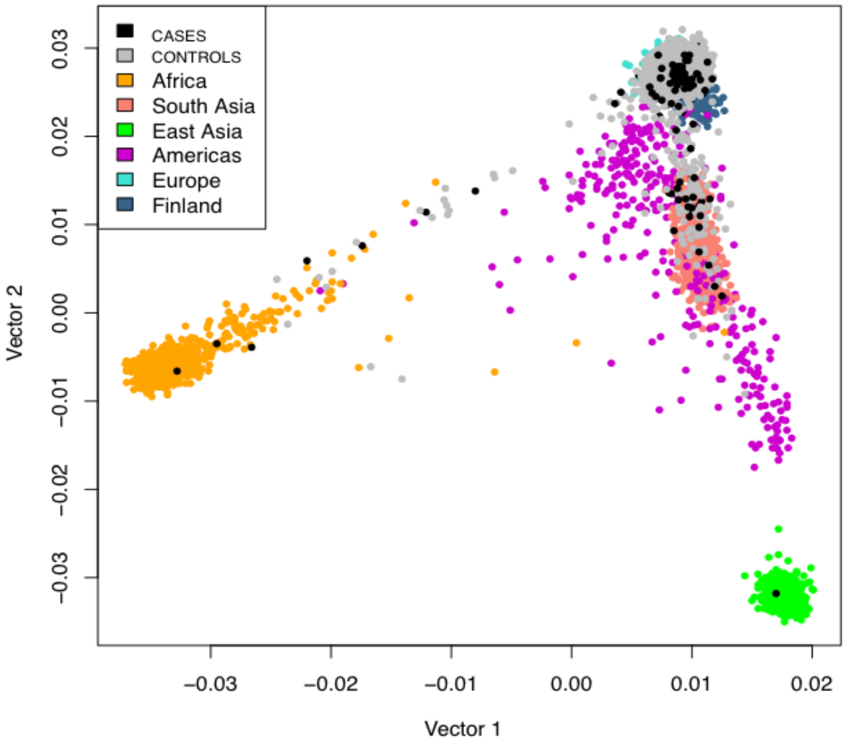

Supplementary Figure 4. Principal components analysis for ancestry inference of VEO-IBD cases and INTERVAL controls. The analysis identified two well defined ethnic clusters of European (104 cases; 3,787 controls) or South Asian ethnicity (21 cases; 68 controls), with the remaining 20 cases and 115 controls scattered throughout the PCA space. 1KG phase 3 samples (N=2,504) were used as reference set.

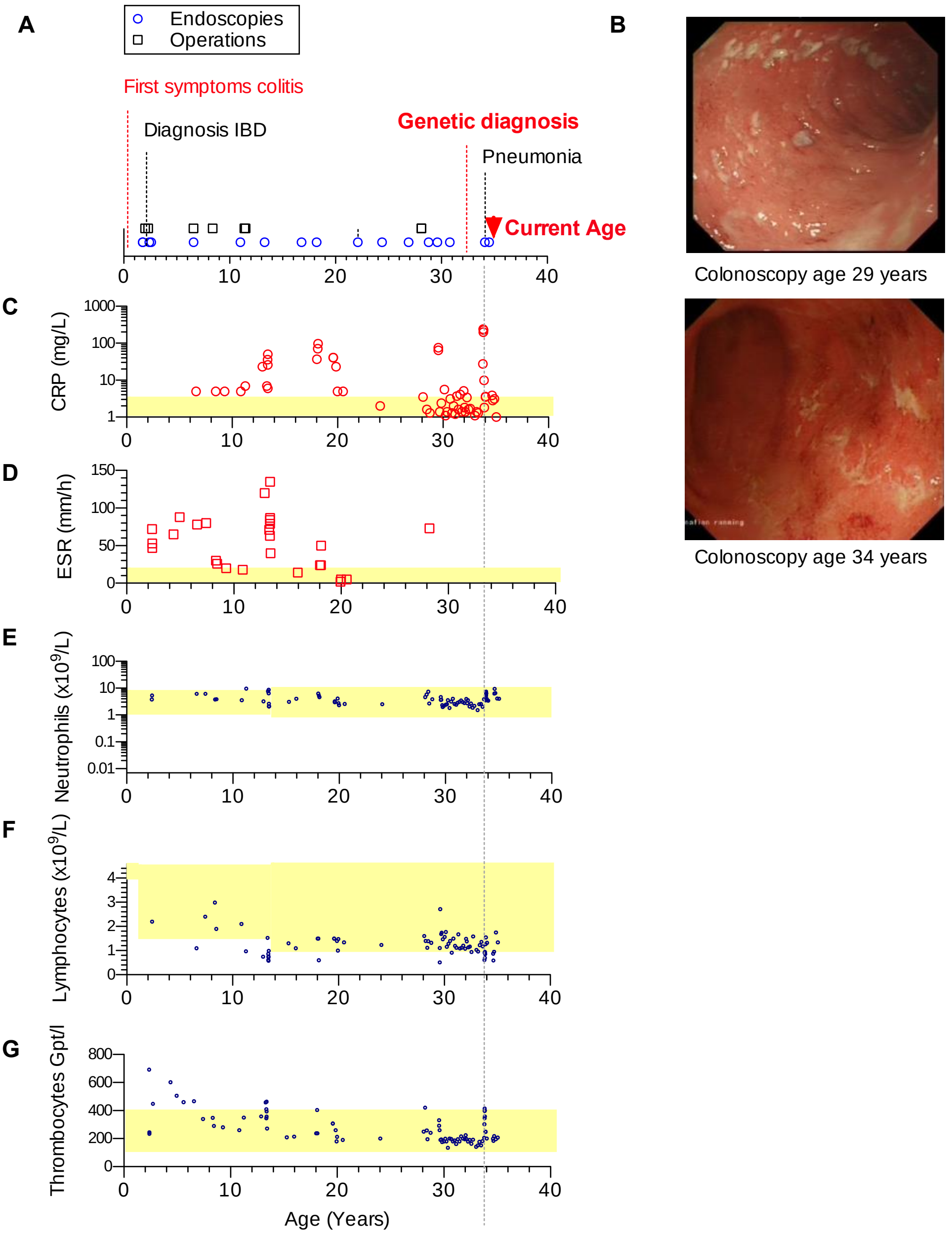

Supplementary Figure 5. Clinical data of the patient with *CYBB* variant mosaicism.

A) Endoscopic investigations and operations over the time of symptoms, endoscopic diagnosis, genetic diagnosis and current age. **B)** Colonoscopic appearance. Example images showing multiple deep ulcerations at 29 and 34 years of age. **C-G)** Haematological and biochemistry inflammatory markers over time. **C)** CRP; **D)** erythrocyte sedimentation rate (ESR), **E)** neutrophil count, **F)** lymphocyte count, and **G)** thrombocyte count. Normal values are indicated by the yellow area. For the analysis C-G all available laboratory parameter that were available in the written and electronic records were collected.

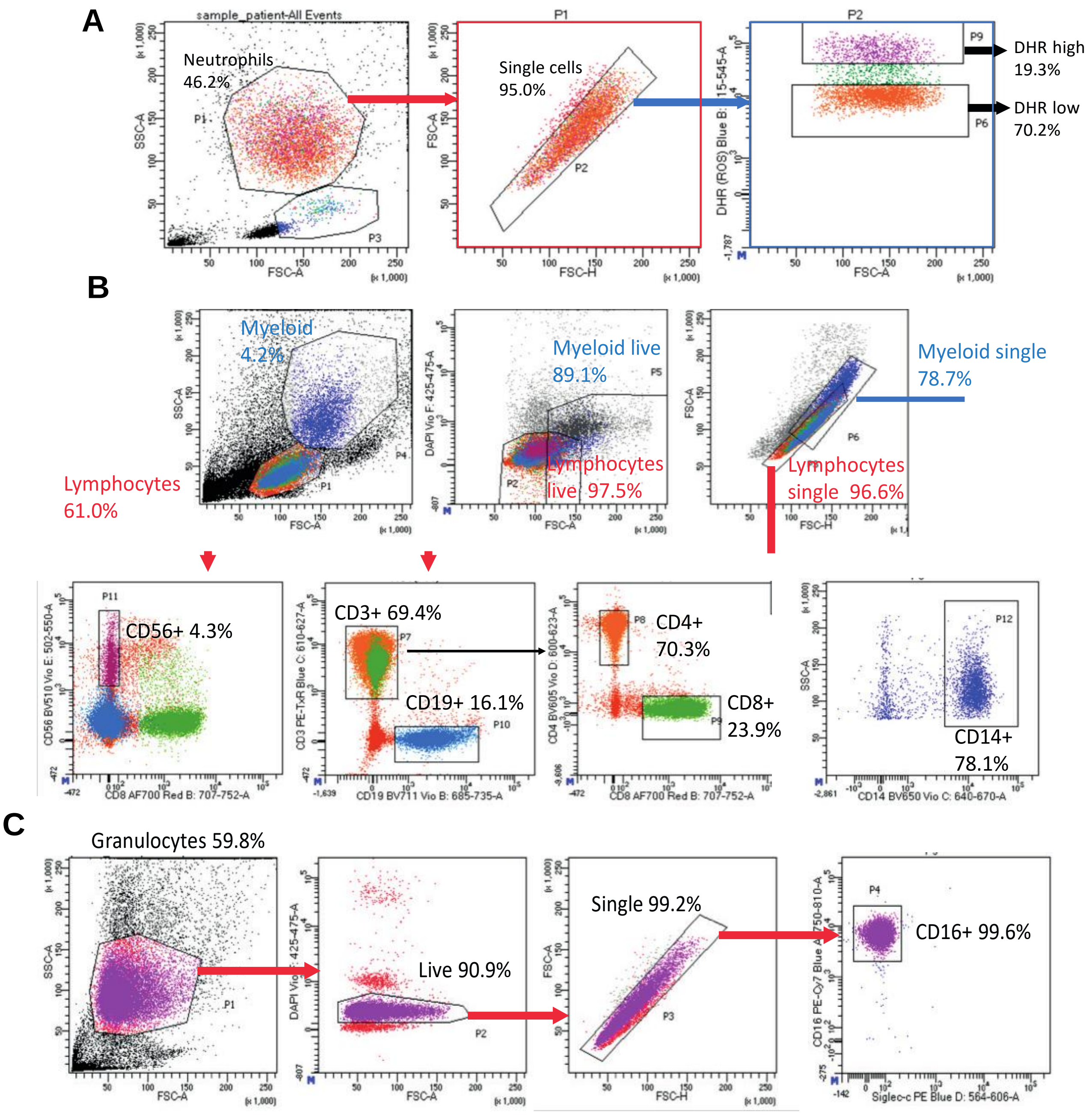

Supplementary Figure 6. FACS strategies for immune cell sorting in the *CYBB* mutant patient. **A)** FACS strategy for cell sorting of DHR-high and DHR-low neutrophil populations following DHR staining and PMA stimulation (presented on Figure 1H). EDTA blood was stained using DHR-123 and stimulated with 50ng/mL PMA. Neutrophils were gated by cell size, followed by single cell gating and lastly gating on DHR high and low cells for the mosaic *CYBB* patient. A similar strategy was followed for the healthy donor; all cells were DHR high in the donor. **B)** FACS strategy for PBMC-sorted populations. PBMCs were isolated from whole blood using Lymphoprep and stained using CD56 (BV510, clone HCD56, Biolegend), CD14 (BV650, clone M5E2, Biolegend), CD19 (BV711,

clone SJ25C1, BD Horizon), CD3 (PE/Dazzle 594, clone UCHT1, Biolegend), CD4 (BV605, clone OKT4, Biolegend), CD8 (AF700, clone SK1, Biolegend) and DAPI. **C)** FACS strategy for CD16+ cell sorting. Granulocytes were stained for CD16 (PE-Cy7, clone 3G8, Biolegend), Siglec-8 (PE, clone 7C9, Biolegend) and DAPI.

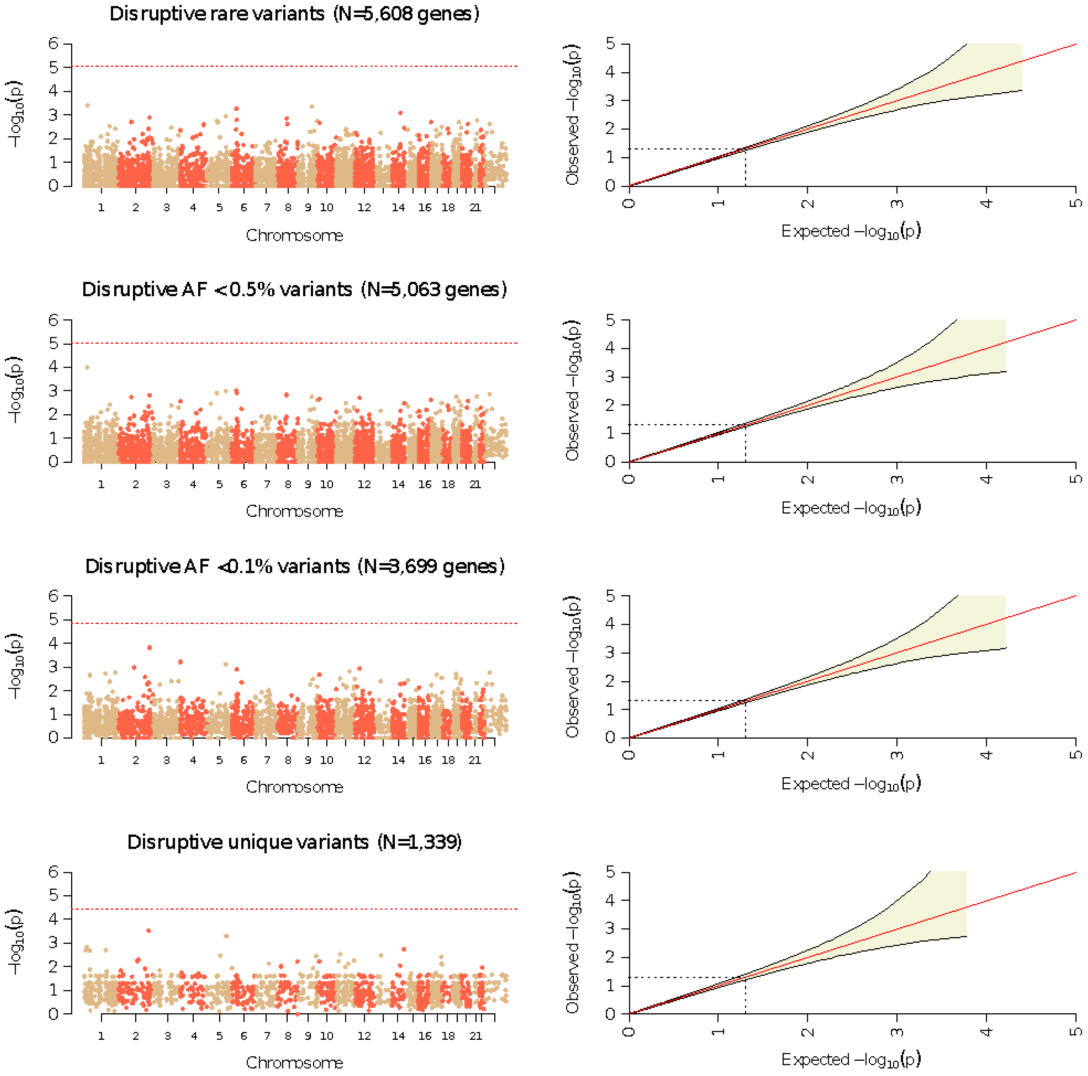

Supplementary Figure 7. Gene-wise burden tests of rare disruptive alleles at four allele frequency thresholds in Europeans and South Asian samples combined (N=124 VEO-IBD cases; 3,855 INTERVAL controls). A) *P*-values (Fisher's exact test) obtained from the gene association tests at various AF thresholds (similar results obtained when running the analysis on all rare variants (i.e. not just disruptive; see Methods for definitions of variant categories). No gene reached exome-wide significance (assuming a cut-off of $P < 1.7\times10^{-6}$ after correction for multiple

testing). **B)** QQ plots of P-values, showing no deviation from the null at the end of the tail. There is no evidence of inflation in the test statistics from the expected distribution, which suggests cases and controls were well matched for potential confounding factors such as ancestry.

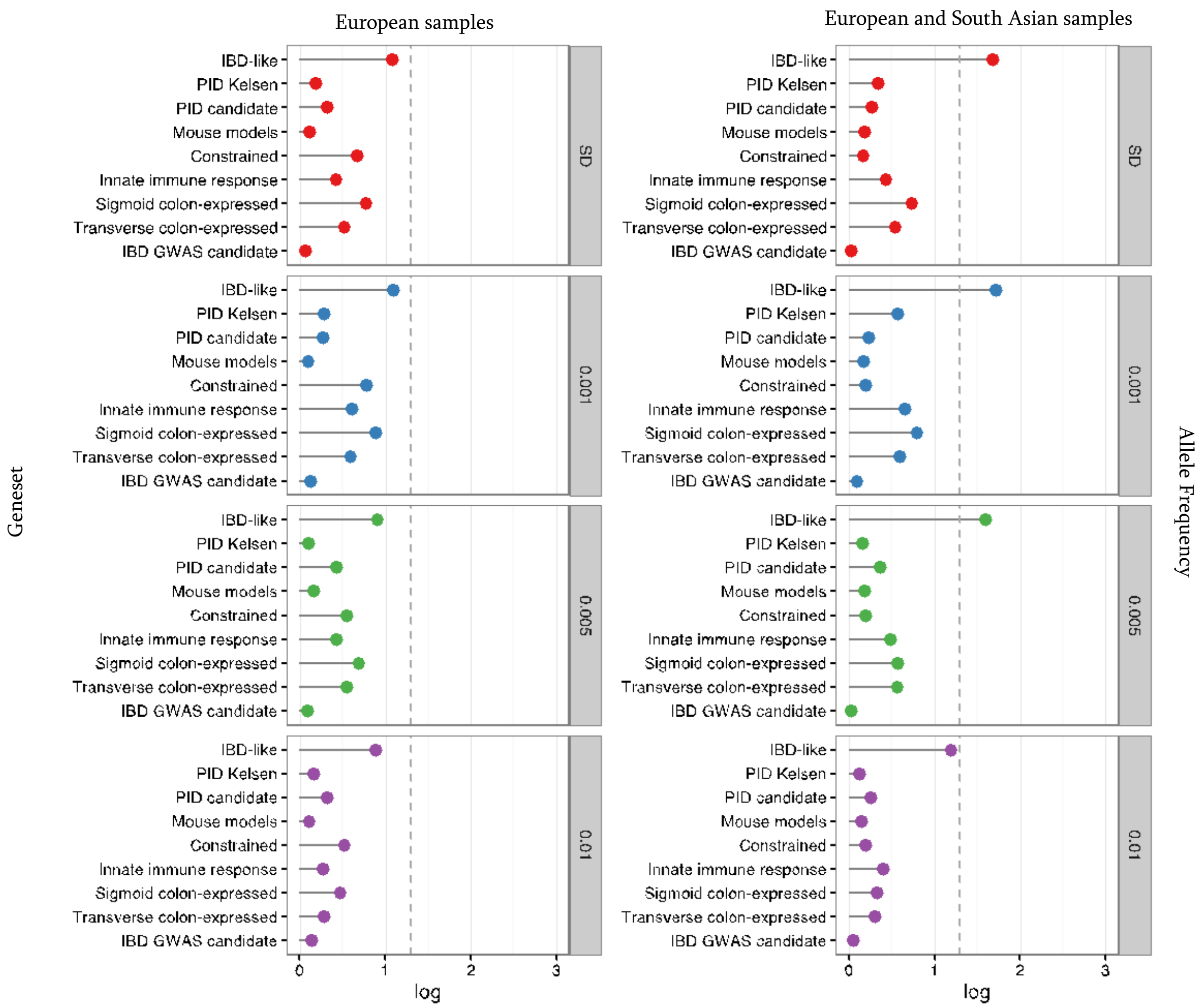

Supplementary Figure 8. Geneset analysis of disruptive variants stratified by allele frequency. The x-axis represents the –log10 of the *P*-value (PLINK/SEQ burden test statistic) of the geneset enrichment relative to the exome-wide enrichment between cases-controls. Dashed vertical line represents the statistical significance at alpha=0.05. *P*-value in figure is uncorrected for multiple testing, but no gene reached the required threshold (*P*=0.0014). The addition of South Asian individuals drove an association signal (P=0.019) for VEO-IBD genes composed of *XIAP*, *TTC37*, *TTC7A* and *CYBA*, but did not withstand correction for multiple testing. No exome-wide differences were observed between cases and controls (i.e. when taking into account all genes in the exome).

"IBD-like" genes refer to the list of 67 genes known to be associated with Mendelian disorders with IBD-like inflammatory phenotypes; for information on the size and component of each geneset see **Supplementary Table 3.**

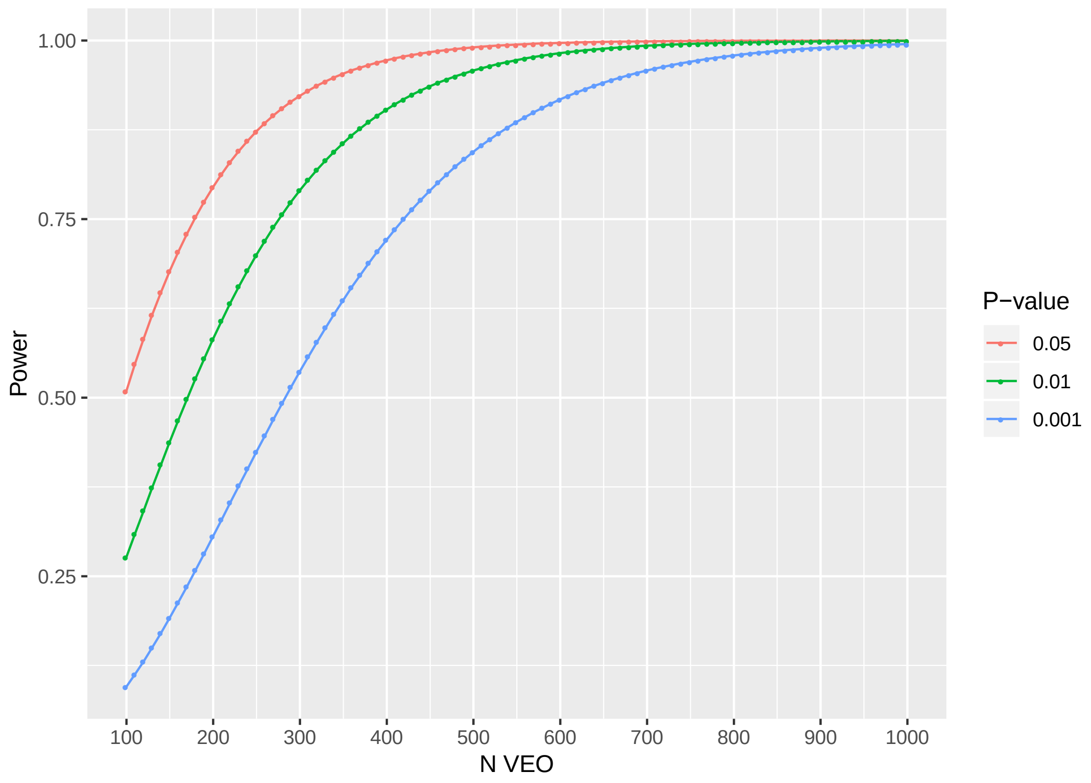

Supplementary Figure 9. Power estimations to detect a Cohen's D effect size of 0.2 between the PRS means of a cohort of 6318 samples (which corresponds to the number of European adult UC cases) and a second cohort of N VEO cases (x axis) using a *t-test*. Similar number of VEO cases would be required to estimate changes in PRS when compared to CD adult cases (N = 7578, data not shown).

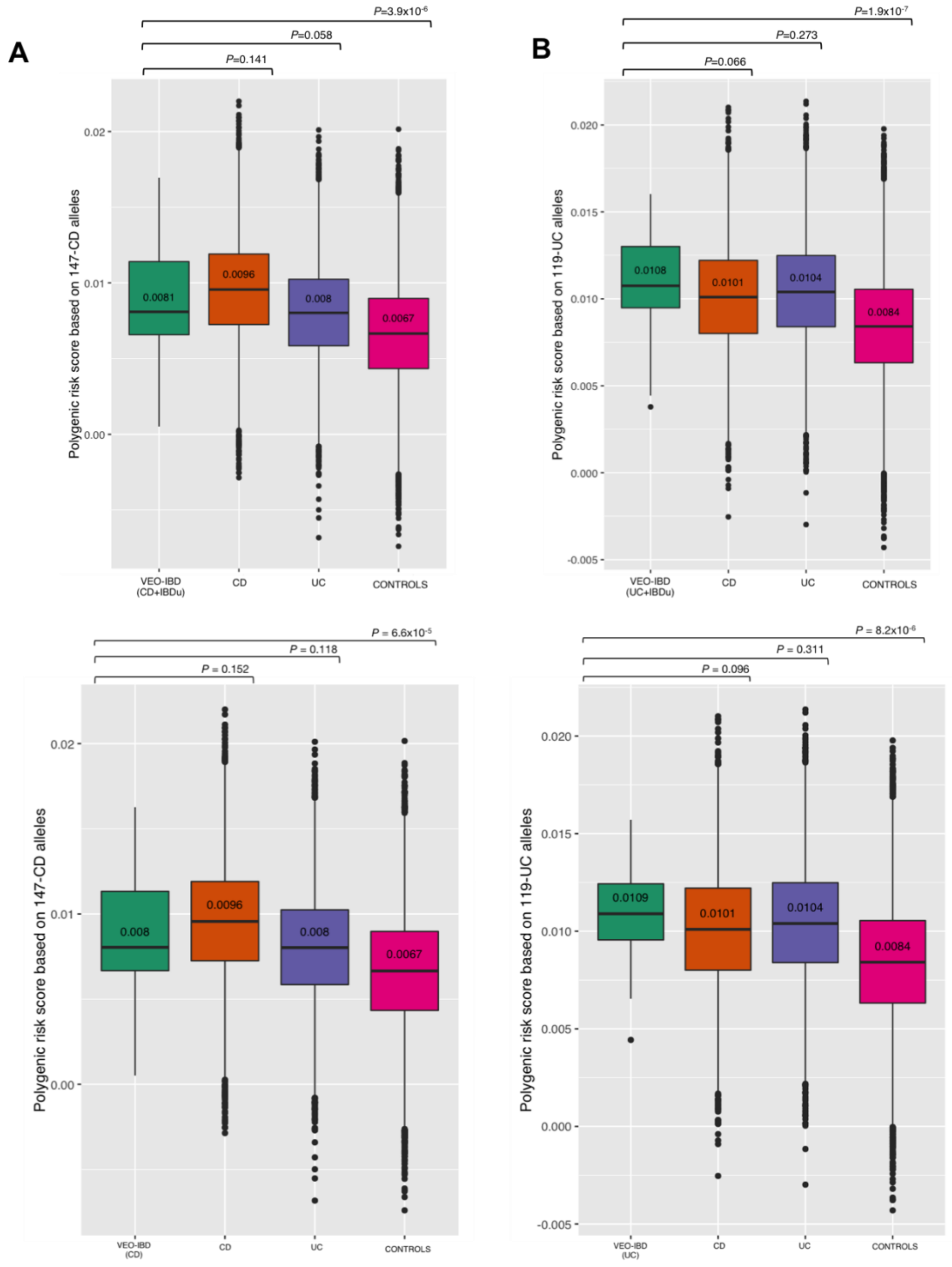

Supplementary Figure 10. Distribution of CD (A) and UC (B) risk scores in VEO-IBD, CD, UC cases and healthy controls. VEO-IBD cases were stratified into CD and UC (bottom row) and into CD+IBDu and UC+IBDu (top row). The CD score was calculated using 147 CD risk alleles and the UC score using 119 UC risk alleles. Both scores were generated for 99 European VEO-IBD cases (with high quality genotyping data), 7,578 CD cases 6,318 UC cases and 18,780 population

controls, all of European ancestry. The analysis in VEO-IBD cases was conducted by restricting the CD burden tests to those VEO-IBD cases defined as CD+IBDu (indeterminate IBD), and the UC burden tests restricted to those with UC+IBDu (indeterminate IBD). VEO-IBD sample size details: 48 CD, 18 IBDu and 33 UC patients. Discovery cohort: COLORS-in-IBD cases (N=99) vs UKIBDGC cases and controls. The Student's t-test was used in group comparisons.

SUPPLEMENTARY TABLES

Baseline patient characteristics		N = 145	
Gender (*female/male*)		63 (43%) / 82 (57%)	
Ethnicity (*Caucasian / Black / Asian / Jewish / Others / unknown)*		99 (68%) / 2 / 21 / 1 / 11 / 11	
Age at diagnosis, years			
• Mean ± SD		3.6 ± 1.7	
• Median (range)		3.5 (range 4 weeks to 6.8 years)	
Diagnosis (*CD / UC / IBDU*)		66 (46%) / 51 (35%) / 28 (19%)	
Positive family history		29/137 (21.2%)	
Paris Crohn's classification (n=66) *			
• Disease location	L1 Ileum	2	
	L2 Colon	20	
	L3 Ileocolonic	42	
	+ L4 (upper GI tract)	34	
• Disease behaviour	B1 nonstricturing-nonpenetrating	54	
	B2 stricturing	6	
	B3 penetrating	2	
	B2B3 penetrating and stricturing	3	
	Perianal involvement	17 (24.2%)	
Paris UC classification		UC (n=49) **	uIBD (n=25) ***
• Disease location	E1 Ulcerative proctitis	1	0
	E2 Left sided UC to splenic flexure	6	0
	E3 Extensive UC to hepatic flexure	4	1
	E4 Pancolitis	38	24
GI surgery			
Colectomy		23/144 (16.0%)	
Associate medical therapy			
	Steroids	93/144 (64.6%)	
	Azathioprine/6-MP	102/144 (70.8%)	
	MTX	11/144 (7.6%)	
	CYA	9/144 (6.3%)	
	Anti-TNFα	51/144 (35.4%)	

Supplementary Table 1. Demographic and phenotypic characteristics of the VEO-IBD cohort.

*****CD: 2/66 patients with oral and perianal CD only. **UC: 1/51 patient unknown location, 1/51 data set incomplete. ***uIBD (undeterminable IBD): 3/28 patients without macroscopic inflammation.

Biological category	Syndrome/disorder	Gene	Inheritance
Epithelial barrier and response defects	Dystrophic epidermolysis bullosa	*COL7A1*	AR
	Kindler syndrome	*FERMT1*	AR
	X-linked ectodermal dysplasia and immunodeficiency	*IKBKG*	X
	TTC7A deficiency	*TTC7A*	AR
	ADAM17 deficiency	*ADAM17*	AR
	Tufting enteropathy	*EPCAM*	AR
	Congenital diarrhea	*SLC9A3*	AR
	Familial diarrhea	*GUCY2C*	AD
	Prostaglandin transporter	*SLCO2A1*	AR
T- and B-cell selection and differentiation defects	Combined variable immunodeficiency (CVID 1)	*ICOS*	AR
	Combined variable immunodeficiency (CVID 8)	*LRBA*	AR
	Combined variable immunodeficiency (CVID 11)	*IL21*	AR
	BACH2 defect	*BACH2*	AD
	CTLA4 deficiency	*CTLA4*	AD
	Agammaglobulinemia	*BTK*	X
	Agammaglobulinemia	*PIK3R1*	AR
	Hyper-IgM syndrome	*CD40LG*	X
	Hyper-IgM syndrome	*AICDA*	AR
	Wiskott–Aldrich syndrome	*WAS*	X
	Wiskott–Aldrich-like syndrome	*ARPC1B*	AR
	Omenn syndrome	*DCLRE1C*	AR
	Atypical SCID	*RAG1*	AR
	Aypical SCID	*ZAP70*	AR
	Atypical SCID	*RAG2*	AR
	Atypical SCID	*IL2RG*	AR
	Atypical SCID	*LIG4*	AR
	Atypical SCID	*ADA*	AR
	Atypical SCID	*CD3γ*	AR
	Hoyeraal Hreidarsson Syndrome	*DKC1*	X
	Hoyeraal Hreidarsson Syndrome	*RTEL1*	AR
	Loeys-Dietz syndrome 1	*TGFBR1*	AD
	Loeys-Dietz syndrome 2	*TGFBR2*	AD
	PTEN hamartoma tumor syndrome	*PTEN*	AD
	Hyper IgE syndrome	*DOCK8*	AR
Hyper- and auto-inflammatory disorders	Mevalonate kinase deficiency	*MVK*	AR
	Phospholipase Cγ2 defects	*PLCG2*	AD
	Familial mediterranean fever	*MEFV*	AR
	Familial hemophagocytic lymphohistiocytosis type 5	*STXBP2*	AR
	X-linked lymphoproliferative syndrome 2 (XLP2)	*XIAP*	X
	X-linked lymphoproliferative syndrome 1 (XLP1)	*SH2D1A*	X
	Syndrome of enterocolitis and autoinflammation	*NLRC4*	AD
	Hermansky-Pudlak syndrome (type 1)	*HPS1*	AR
	Hermansky-Pudlak syndrome (type 4)	*HPS4*	AR

	Hermansky-Pudlak syndrome (type 6)	*HPS6*	AR
Regulatory T-cells and immune regulation	IPEX	*FOXP3*	X
	IPEX-like	*IL2RA*	AR
	IPEX-like	*STAT1*	AD
	IPEX-like	*MALT1*	AR
	IPEX-like	*STAT3*	AD
	IL-10 signalling defects	*IL10RA*	AR
	IL-10 signalling defects	*IL10RB*	AR
	IL-10 signalling defects	*IL10*	AR
Phagocyte defects	Chronic granulomatous disease	*CYBB*	X
	Chronic granulomatous disease	*CYBA*	AR
	Chronic granulomatous disease	*NCF1*	AR
	Chronic granulomatous disease	*NCF2*	AR
	Chronic granulomatous disease	*NCF4*	AR
	Glycogen storage disease type 1b	*SLC37A4*	AR
	Congenital neutropenia	*G6PC3*	AR
	Leukocyte adhesion deficiency 1	*ITGB2*	AR
	TRIM22 defect	*TRIM22*	AR
	Niemann Pick type C	*NPC1*	AR
Others	MASP2-deficiency	*MASP2*	AR
	Trichohepatoenteric syndrome	*SKIV2L*	AR
	Trichohepatoenteric syndrome	*TTC37*	AR
	Proprotein convertase 1 defect	*PCSK1*	AR
	X-linked reticulate pigmentary disorder	*POLA1*	X

Supplementary Table 2. Genetic defects associated with IBD-inflammatory phenotypes.

Genetic defects are grouped according to functional category. Gene names refer to HUGO gene nomenclature. AR: autosomal recessive; AD: autosomal dominant; X: X-linked disease. Genes were selected on basis of two literature reviews[6,7].

Geneset	Number of genes in set
VEO-IBD and IBD-like inflammation genes	67
Known PID genes	400
Candidate PID genes	6,220
Mouse models of colitis	143
Top 10% constrained genes in the genome	3,289
Known innate immune response genes	165
Sigmoid-colon expressed genes	100
Transverse-colon expressed genes	100
IBD GWAS candidate genes	354

Supplementary Table 3. Genesets tested for a rare variant enrichment in VEO-IBD patients. Genesets were collated from the following sources: the VEO-IBD genes were selected from two recent literature reviews[6,7]; Known PID genes were taken from Kelsen et al 2015[8] and are the same 400 genes tested in their manuscript, consisting of a panel of known PID genes and related pathways; Candidate PID genes were selected from Itan and Casanova 2015[9]; Mouse models of colitis were retrieved from Mizoguchi and Mizoguchi 2010[10]; Top 10% constrained genes in the genome were retrieved from Samocha et al 2014[11]; Known innate immune response genes were retrieved from the InnateDB (www.innatedb.com); Sigmoid and transverse-colon expressed genes were selected from GTEx (www.gtexportal.org); IBD GWAS candidate genes were retrieved from Jostins et al 2012[12] and Liu et al 2015[13].

Baseline patient characteristics	N = 117
Gender (*female/male*)	50 (43%) / 67 (57%)
Ethnicity (Caucasian*)*	117 (100%)
Age at diagnosis, years	
• Mean ± SD	4.43 ± 1.8
• Median (range)	4.89 (range 0.36 to 6.97 years)
Diagnosis (*CD / UC / IBDU*)	40 (34%) / 55 (47%) / 22 (19%)

Supplementary Table 4. Demographic and phenotypic characteristics of the Toronto VEO-IBD cohort. Dataset used in the replication of the PRS scores.

SUPPLEMENTARY REFERENCES

1. Hauck, F. *et al.* Diagnostic and treatment options for severe IBD in female X-CGD carriers with non-random X-inactivation. *J. Crohn's Colitis* **10,** 112–115 (2016).

2. Battersby, A. C., Cale, C. M., Goldblatt, D. & Gennery, A. R. Clinical manifestations of disease in X-linked carriers of chronic granulomatous disease. *J. Clin. Immunol.* **33,** 1276–1284 (2013).

3. Dhillon, S. S. *et al.* Variants in nicotinamide adenine dinucleotide phosphate oxidase complex components determine susceptibility to very early onset inflammatory bowel disease. *Gastroenterology* **147,** 680–689 (2014).

4. Romberg, N. *et al.* Mutation of NLRC4 causes a syndrome of enterocolitis and autoinflammation. *Nat. Genet.* **46,** 1135–1139 (2014).

5. Canna, S. W. *et al.* An activating NLRC4 inflammasome mutation causes autoinflammation with recurrent macrophage activation syndrome. *Nat. Genet.* **46,** 1140–6 (2014).

6. Uhlig, H. H. Monogenic diseases associated with intestinal inflammation: implications for the understanding of inflammatory bowel disease. *Gut* **62,** 1795–1805 (2013).

7. Uhlig, H. H. *et al.* The Diagnostic Approach to Monogenic Very Early Onset Inflammatory Bowel Disease. *Gastroenterology* **147,** 990–1007.e3 (2014).

8. Kelsen, J. R. *et al.* Exome Sequencing Analysis Reveals Variants in Primary Immunodeficiency Genes in Patients With Very Early Onset Inflammatory Bowel Disease. *Gastroenterology* **149,** 1415–1424 (2015).

9. Itan, Y. & Casanova, J.-L. Novel primary immunodeficiency candidate genes predicted by the human gene connectome. *Front. Immunol.* **6,** 142 (2015).

10. Mizoguchi, A. & Mizoguchi, E. Animal models of IBD: Linkage to human disease. *Curr. Opin. Pharmacol.* **10,** 578–587 (2010).

11. Samocha, K. E. *et al.* A framework for the interpretation of de novo mutation in human disease. *Nat. Genet.* **46,** 944–50 (2014).

12. Jostins, L. *et al.* Host–microbe interactions have shaped the genetic architecture of inflammatory bowel disease. *Nature* **491,** 119–124 (2012).

13. Liu, J. Z. *et al.* Association analyses identify 38 susceptibility loci for inflammatory bowel disease and highlight shared genetic risk across populations. *Nat. Genet.* **47,** 979 (2015).
