# Supplementary material for: Somatic mosaicism and common genetic variation contribute to the risk of very-early-onset inflammatory bowel disease
Source: Nat Commun. 2020 Feb 21;11:995. doi: 10.1038/s41467-019-14275-y (PMC7035382; doi:10.1038/s41467-019-14275-y)
Supplement: Supplementary file 3 — Reporting Summary [file 41467_2019_14275_MOESM3_ESM.pdf]

nature research

Corresponding author(s): Holm H. Uhlig and Carl A. Anderson

Last updated by author(s): Jul 14, 2019

Reporting Summary

Nature Research wishes to improve the reproducibility of the work that we publish. This form provides structure for consistency and transparency in reporting. For further information on Nature Research policies, see Authors & Referees and the Editorial Policy Checklist.

Statistics

For all statistical analyses, confirm that the following items are present in the figure legend, table legend, main text, or Methods section.

n/a	Confirmed	
☐	☒	The exact sample size (*n*) for each experimental group/condition, given as a discrete number and unit of measurement
☐	☒	A statement on whether measurements were taken from distinct samples or whether the same sample was measured repeatedly
☐	☒	The statistical test(s) used AND whether they are one- or two-sided<br>*Only common tests should be described solely by name; describe more complex techniques in the Methods section.*
☐	☒	A description of all covariates tested
☐	☒	A description of any assumptions or corrections, such as tests of normality and adjustment for multiple comparisons
☐	☒	A full description of the statistical parameters including central tendency (e.g. means) or other basic estimates (e.g. regression coefficient) AND variation (e.g. standard deviation) or associated estimates of uncertainty (e.g. confidence intervals)
☐	☒	For null hypothesis testing, the test statistic (e.g. *F*, *t*, *r*) with confidence intervals, effect sizes, degrees of freedom and *P* value noted<br>*Give P values as exact values whenever suitable.*
☐	☒	For Bayesian analysis, information on the choice of priors and Markov chain Monte Carlo settings
☐	☒	For hierarchical and complex designs, identification of the appropriate level for tests and full reporting of outcomes
☐	☒	Estimates of effect sizes (e.g. Cohen's *d*, Pearson's *r*), indicating how they were calculated

Our web collection on statistics for biologists contains articles on many of the points above.

Software and code

Policy information about availability of computer code

Data collection	Not applicable in terms of data collection
Data analysis	Available in detailed Methods section, along with versions used. Briefly, NGS data was processed using BWA, GATK Haplotype caller, verifyBAMID, VQSR, SAMTOOLS, PLINK/SEQ (all open source). Genotype data was processed using SHAPEIT, IMPUTE and PLINK (all open source).

For manuscripts utilizing custom algorithms or software that are central to the research but not yet described in published literature, software must be made available to editors/reviewers. We strongly encourage code deposition in a community repository (e.g. GitHub). See the Nature Research guidelines for submitting code & software for further information.

Data

Policy information about availability of data

All manuscripts must include a data availability statement. This statement should provide the following information, where applicable:

- Accession codes, unique identifiers, or web links for publicly available datasets
- A list of figures that have associated raw data
- A description of any restrictions on data availability

Sequencing and genotyping data that supports this study have been deposited to the European Genome-phenome Archive (EGA) under the accession code EGAS00001000513 and EGAS00001000924, respectively.

Field-specific reporting

Please select the one below that is the best fit for your research. If you are not sure, read the appropriate sections before making your selection.

☒ Life sciences ☐ Behavioural & social sciences ☐ Ecological, evolutionary & environmental sciences

For a reference copy of the document with all sections, see nature.com/documents/nr-reporting-summary-flat.pdf

Life sciences study design

All studies must disclose on these points even when the disclosure is negative.

Sample size	146 VEO-IBD cases and 4436 INTERVAL controls were sequenced and analysed in this study. 99 VEO-IBD cases out of the 146 were also genotyped and compared to a cohort of 18,780 genotyped controls, followed by a replication experiment in further 117 VEO-IBD cases and 2,603 controls from other independent cohorts.
Data exclusions	Explained in detail in Methods section. Quality control was performed on both cases and controls, in both sequencing and genotyping data.
Replication	Polygenic risk scores were replicated in an independent cohort of VEO-IBD cases and controls. Power calculations were also conducted.
Randomization	Not relevant to this study. Sample groups include cases and controls solely.
Blinding	Not relevant to this study. Sample groups include cases and controls solely.

Reporting for specific materials, systems and methods

We require information from authors about some types of materials, experimental systems and methods used in many studies. Here, indicate whether each material, system or method listed is relevant to your study. If you are not sure if a list item applies to your research, read the appropriate section before selecting a response.

Materials & experimental systems

n/a	Involved in the study	
☐	☒	Antibodies
☒	☐	Eukaryotic cell lines
☒	☐	Palaeontology
☒	☐	Animals and other organisms
☐	☒	Human research participants
☒	☐	Clinical data

Methods

n/a	Involved in the study	
☒	☐	ChIP-seq
☐	☒	Flow cytometry
☒	☐	MRI-based neuroimaging

Antibodies

Antibodies used	C-terminal antibody to measure SAP expression (SH2D1A, Stratech Scientific Biosciences; clone 1C9, cat H00004068-MO1) or isotype control antibody (IgG1 isotype control; BD Biosciences 349040). Samples were again washed and stained with anti-mouse IgG1-FITC (Dako; F0479) before FACS analysis.<br>Immune cells were sorted on CD56 (BV510, clone HCD56, Biolegend, catalog number 318340), CD14 (BV650, clone M5E2, Biolegend, catalog number 563420), CD19 (BV711, clone SJ25C1, BD Horizon, catalog number 563038), CD3 (PE/Dazzle 594, clone UCHT1, Biolegend, catalog number 300450), CD4 (BV605, clone OKT4, Biolegend, catalog number 317438), CD8 (AF700, clone SK1, Biolegend, catalog number 344724) and DAPI (1:8000 dilution). To stain the granulocytes, CD16 (PE-Cy7, clone 3G8, Biolegend, catalog number 302016), Siglec-8 (PE, clone 7C9, Biolegend, catalog number 347104) and DAPI (1:8000 dilution) were used. All antibodies were used at a 1:100 dilution unless otherwise stated. Cells were washed and filtered prior to sorting on BD FACS Aria III. For gp91 staining, we used anti-flavachrome b558 (7D5 gp91) FITC antibody (MBL, cat. No. D162-4) or mouse IgG1 FITC (BD Biosciences, cat. No. M075-4). For DHR FACS, DHR-123 (Life Technologies, D23806) was used at a final concentration of 2.5µg/ml.
Validation	*Describe the validation of each primary antibody for the species and application, noting any validation statements on the manufacturer's website, relevant citations, antibody profiles in online databases, or data provided in the manuscript.*

Human research participants

Policy information about studies involving human research participants

Population characteristics	Described in detail in Supplementary Table 1. Briefly, VEO-IBD cases had a median age at diagnosis of 3.5yrs (range from 4 weeks to 6.8 yrs), 43% were female, 46% diagnosed with CD, 35% diagnosed with UC and the remaining with undeterminable IBD. 21% with a positive family history.

Recruitment	Described in detail in Methods. Samples were referred from participating centres in the United Kingdom, Switzerland, Poland and Germany. All patients had a confirmed diagnosis of IBD by standard methods, including endoscopic, radiologic, laboratory, and clinical evaluation (ESPGHAN guidelines44). Phenotypic status was based on the Paris Classification45. Patients were selected according to age-at-diagnosis (< 7 years, age of symptom onset < 6 years) and the severity of the IBD phenotype, as indicated by need for surgery and/or therapy progression to biologics or immunomodulators. When a clinical diagnosis of a known Mendelian disease was suspected (e.g., IL10, IL10RB or IL10RA defects in patients with IBD onset in the first three months of life), candidate genes were pre-screened by a clinical genetics laboratory. If a genetic diagnosis was established, the individual was excluded from our study in an attempt to enrich the cohort with cases harbouring undiscovered monogenic causes of VEO-IBD. Detailed demographic and phenotypic characteristics of the VEO-IBD cohort are provided in Supplementary Table 1.
Ethics oversight	The study was approved by the North Staffordshire Research Ethics Committee (REC: 09/H1204/30; subproject COLORS in IBD) and local ethics committees at the study sites. All patients, or their parents, gave written informed consent before enrolment.

Note that full information on the approval of the study protocol must also be provided in the manuscript.

Flow Cytometry

Plots

Confirm that:

☒ The axis labels state the marker and fluorochrome used (e.g. CD4-FITC).

☒ The axis scales are clearly visible. Include numbers along axes only for bottom left plot of group (a 'group' is an analysis of identical markers).

☒ All plots are contour plots with outliers or pseudocolor plots.

☒ A numerical value for number of cells or percentage (with statistics) is provided.

Methodology

Sample preparation	The neutrophil oxidative burst assay to detect reactive oxygen species by DHR FACS assay was performed using standard techniques. Briefly, EDTA blood was incubated with DHR-123 for 15 min at 37°C followed by PMA (100ng/ml) stimulation and FACS staining. DHR response was measured in FSC/SCC gated neutrophils. The stimulation index refers to the ratio of the mean fluorescence of the stimulated cells to the mean fluorescence observed in the unstimulated cells in the DHR assay. For the sorting of different immune populations, PBMCs were isolated from whole blood using Lymphoprep (Axis-Shieldand) and Ficoll gradient centrifugation. Cells were re-suspended in RPMI-1640 (Sigma), the granulocyte layer extracted65, erythrocytes lysed with water and the white granulocyte pellet was collected. Cells were stained using CD56 (BV510, clone HCD56, Biolegend), CD14 (BV650, clone M5E2, Biolegend), CD19 (BV711, clone SJ25C1, BD Horizon), CD3 (PE/Dazzle 594, clone UCHT1, Biolegend), CD4 (BV605, clone OKT4, Biolegend), CD8 (AF700, clone SK1, Biolegend) and DAPI. To stain the granulocytes, CD16 (PE-Cy7, clone 3G8, Biolegend), Siglec-8 (PE, clone 7C9, Biolegend) and DAPI were used. Cells were washed and filtered prior to sorting on a BD FACS Aria III. DNA of the HEK293T (ATCC-CRL-11268) cell line was used as a control.<br>For gp91 staining: We used 500uL EDTA blood to assess gp91 in patient cells. The antibodies used were anti-flavachrome b558 (7D5 gp91) FITC antibody (MBL, cat. No. D162-4) or mouse IgG1 FITC (BD Biosciences, cat. No. M075-4) and were incubated with the samples for 10 minutes at room temperature. FACS lyse (BD Pharmingen, cat no 349202) was added for 10 minutes, followed by cell fix (BD Biosciences, cat no 640181). Cells were acquired on FACSCalibur.
Instrument	BD FACS Aria III for sorting and BD Fortessa for flow cytometry or FACSCalibur.
Software	FlowJo was used to analyze the results.
Cell population abundance	Provided in Supplementary Figure 6
Gating strategy	Provided in Supplementary Figure 6

☒ Tick this box to confirm that a figure exemplifying the gating strategy is provided in the Supplementary Information.
